# Age Differences in Encoding-Related Alpha Power Reflect Sentence Comprehension Difficulties

**DOI:** 10.3389/fnagi.2019.00183

**Published:** 2019-07-17

**Authors:** Caroline Beese, Benedict Vassileiou, Angela D. Friederici, Lars Meyer

**Affiliations:** ^1^Department of Neuropsychology, Max Planck Institute for Human Cognitive and Brain Sciences, Leipzig, Germany; ^2^Research Group Language Cycles, Max Planck Institute for Human Cognitive and Brain Sciences, Leipzig, Germany

**Keywords:** aging, alpha band, encoding, neural oscillations, sentence comprehension

## Abstract

When sentence processing taxes verbal working memory, comprehension difficulties arise. This is specifically the case when processing resources decline with advancing adult age. Such decline likely affects the encoding of sentences into working memory, which constitutes the basis for successful comprehension. To assess age differences in encoding-related electrophysiological activity, we recorded the electroencephalogram from three age groups (24, 43, and 65 years). Using an auditory sentence comprehension task, age differences in encoding-related oscillatory power were examined with respect to the accuracy of the given response. That is, the difference in oscillatory power between correctly and incorrectly encoded sentences, yielding subsequent memory effects (SME), was compared across age groups. Across age groups, we observed an age-related SME inversion in the alpha band from a power decrease in younger adults to a power increase in older adults. We suggest that this SME inversion underlies age-related comprehension difficulties. With alpha being commonly linked to inhibitory processes, this shift may reflect a change in the cortical inhibition–disinhibition balance. A cortical disinhibition may imply enriched sentence encoding in younger adults. In contrast, resource limitations in older adults may necessitate an increase in cortical inhibition during sentence encoding to avoid an information overload. Overall, our findings tentatively suggest that age-related comprehension difficulties are associated with alterations to the electrophysiological dynamics subserving general higher cognitive functions.

## Introduction

Sentence comprehension remains generally well-preserved across the adult lifespan (Shafto and Tyler, 2014). However, when sentence processing taxes verbal working memory (vWM), comprehension difficulties arise, in particular with advancing adult age (e.g., Feier and Gerstman, 1980; Kemper, 1986; Obler et al., 1991), that is, when vWM capacity declines (e.g., Bopp and Verhaeghen, 2005). One prerequisite for accurate sentence comprehension is the successful encoding of sentences into vWM as sentences unfold. Age-related comprehension difficulties may reflect an inefficiency in old age to encode sentences into vWM (Friedman and JohnsonJr., 2014). Here, we examined this hypothesis by comparing the neural correlates of sentence encoding, indirectly indicated by comprehension accuracy, across the lifespan.

While substantial behavioral evidence on age differences in vWM-taxing sentence comprehension shows lower accuracy and longer response times in older than younger adults (e.g., Kemper, 1986; Obler et al., 1991; Kemper et al., 2004), there are only few studies on age differences in the electrophysiological correlates of sentence comprehension (e.g., Gunter et al., 2002; Alatorre-Cruz et al., 2018). Previously, age differences in both syntactic and semantic processing have been related to altered event-related potentials (ERP). While ERPs related to early syntactic processes have been found age-invariant, those related to later syntactic processes have been shown compromised. For example, under high vWM load, compromised processing of the agreement of syntactic features resulted in a diminished left anterior negativity and P600 in older compared to younger adults (Alatorre-Cruz et al., 2018). But even when sentence material is not specifically vWM-taxing, the P600 is diminished and delayed, for instance, in response to phrase structure violations (Friederici et al., 2002 in: Gunter et al., 2002). This may indicate more general syntactic processing difficulties in older adults (e.g., Osterhout and Holcomb, 1992; Gibson, 1998; Kaan et al., 2000; Fiebach et al., 2001). Like the P600, the N400 component, which is associated with semantic processing (Kutas and Hillyard, 1980, 1984; Van Berkum et al., 1999, 2005), has been shown to be diminished and delayed in older compared to younger adults (e.g., Gunter et al., 1992; Kutas and Iragui, 1998; Federmeier et al., 2002; Wlotko et al., 2012). Overall, compared to younger adults, older adults exhibit lower ERP amplitudes and longer latencies during sentence comprehension (Gunter et al., 1992; Kutas and Iragui, 1998; e.g., Federmeier et al., 2002; Friederici et al., 2002 in: Gunter et al., 2002; Wlotko et al., 2012).

While ERP evidence indicates detrimental effects of aging on vWM-taxing sentence comprehension, ERPs are limited to the assessment of evoked (i.e., time– and phase-locked) neural activity. In contrast, here we scrutinized on oscillatory power changes that reflect both evoked and induced electrophysiological dynamics (Sarnthein et al., 1998; Pfurtscheller and Lopes da Silva, 1999). In general, oscillatory power changes within the theta (4–8 Hz), alpha (8–12 Hz), and beta (15–30 Hz) frequency bands have been related to sentence comprehension (e.g., Bastiaansen and Hagoort, 2015; Ding et al., 2016; for review see Meyer, 2017). Syntactic processing and specifically syntactic integration of single words into sentences has been related to increased theta-band power (e.g., Bastiaansen et al., 2002a, 2009; Haarmann and Cameron, 2005; Meyer et al., 2015). In comparison, a decrease in alpha-band power has been associated with vWM encoding of syntactic information (Bastiaansen et al., 2009; Vassileiou et al., 2018), while an increase in alpha-band power has been associated with vWM storage of syntactic information (e.g., Haarmann and Cameron, 2005; Weiss et al., 2005; Meyer et al., 2013; Bonhage et al., 2017). Finally, a decrease in beta-band power has been linked to semantic and predictive processing (e.g., Weiss and Rappelsberger, 1996; Wang et al., 2012; Lewis and Bastiaansen, 2015). Yet, the hypothesis that age-related sentence comprehension difficulties may be reflected by changes to oscillatory dynamics has not been pursued thus far.

We hypothesized here that age-related sentence comprehension difficulties may arise from a sentence encoding inefficiency at old age (Friedman and JohnsonJr., 2014). Therefore, in the current study, we investigated age differences in the oscillatory dynamics underlying sentence encoding. The quantification of oscillatory activity during successful encoding was based on encoding behavior. Specifically, we compared the difference in oscillatory power between later correctly comprehended, or later-remembered (LR), sentences, and later incorrectly comprehended, or later-not-remembered (LNR), sentences. This difference constitutes the subsequent memory effect (SME; Paller et al., 1987; Paller and Wagner, 2002). SMEs reflect oscillatory dynamics that index the accessibility of the information that needs to be encoded for accurate sentence comprehension. SME paradigms are thus well suited to investigate a possible link between age-related sentence comprehension difficulties and sentence encoding inefficiency. Prior literature from memory research describes positive SMEs (i.e., encoding-related oscillatory power of LR > LNR) in the theta band (e.g., Karrasch et al., 2004; Osipova et al., 2006; Friese et al., 2013) and negative SMEs (i.e., encoding-related oscillatory power of LR < LNR) in the alpha and beta bands in younger adults (e.g., Otten et al., 2001; Schott et al., 2002; Hanslmayr and Staudigl, 2014). The effects of healthy aging on SMEs are controversial, including age-invariant SMEs (e.g., Shing et al., 2016), SMEs only in younger but not in older adults (Friedman et al., 1996; Friedman and Trott, 2000; Kamp and Zimmer, 2015), and attenuated or even inverted SMEs in older compared to younger adults (Maillet and Rajah, 2014; Mattson et al., 2014; de Chastelaine et al., 2015). However, none of these studies probed the encoding of entire sentences. It remains an open question whether age-related differences in encoding-related oscillatory neural activity associate with age-related sentence comprehension difficulties.

We hypothesized that age differences in successful sentence encoding are reflected by specific age differences in the encoding-related oscillatory power. To this end, we compared the oscillatory power of correctly and incorrectly encoded sentences, as indicated by sentence comprehension accuracy, between younger, middle-aged, and older adults. As previous studies showed encoding inefficiency already around midlife (Cansino et al., 2010) but comprehension difficulties only after midlife (Sommers, 2015), it was crucial to include the group of middle-aged adults. We expected vWM limitations in older adults to associate with attenuated or even inverted SMEs compared to younger adults. Specifically, we hypothesized to find a positive theta-band SME (Klimesch, 1999) and negative alpha– and beta-band SMEs (Hanslmayr and Staudigl, 2014) in younger adults. Our results confirmed that the negative alpha-band SME in younger adults is attenuated in middle-aged adults and inverted in older adults. This may indicate a decreased encoding efficiency associated with age-related sentence comprehension difficulties.

## Materials and Methods

### Participants

An original sample of 59 healthy right-handed native speakers of German, divided into three age groups (20 younger, 19 middle-aged and 20 older adults), participated in this study. After excluding below-chance performers (see *Statistical analysis*), data from 18 younger adults (8 male; mean age = 24.39 years; *SD* = 1.30 years), 16 middle-aged adults (8 male; mean age = 42.50 years; *SD* = 2.22 years) and 13 older adults (5 male; mean age = 64.00 years; *SD* = 2.45 years) were included into the statistical analysis. The age groups were matched for their level of education (≥14 years of education). All participants had normal or corrected-to-normal vision. No participant suffered from any hearing loss (hearing threshold ≤25 dB as assessed by standard audiometry, Oscilla^®^ SM910-B, Aarhus, Denmark). No participant was demented (Mini Mental State Examination 2 Score ≥27; Folstein et al., 2010) or reported any other neurological disease. Prior to the experiment, all participants gave written informed consent. The study was approved by the ethics committee of the University of Leipzig and was conducted in accordance with the Declaration of Helsinki.

### Standardized Neuropsychological Measures

Sentence processing has been shown supported by several domain-general cognitive functions (e.g., Fedorenko, 2014). To assess possible relations to task-related domain-general cognitive functions, all participants were administered a neuropsychological test battery, measuring working memory (Non-word Repetition Span: Welte, 1981; Counting Span Task: Case et al., 1982; Digit Span Forward/Backward: Aster et al., 2006), auditory attention (Auditory Flankers Task; Chan et al., 2005), verbal intelligence (Similarities and Vocabulary Task), and non-verbal intelligence (Matrices and Block Tasks; Aster et al., 2006). Table 1 offers an exhaustive overview of age group differences across all neuropsychological test measures.

**TABLE 1:** Demographics and individual differences (*M* = mean, *SD* = standard deviation).

**Age Group**							**Statistics**	
	**Young (*n* = 18)**		**Middle (*n* = 16)**		**Old (*n* = 13)**		***F*(2,44)**	***p***
**Measure**	***M***	***SD***	***M***	***SD***	***M***	***SD***		
**Demographics**								
Age	24.39	1.30	42.50	2.22	64.00	2.45		
Education	16.83	1.50	19.88	3.13	18.38	2.54	6.56	3.2 × 10^–3^
**Verbal Intelligence**								
Vocabulary Task	53.28	4.76	53.94	5.01	50.00	5.86	2.34	0.11
Similarities Task	27.22	3.25	27.88	3.26	27.69	2.72	0.20	0.83
Composite	0.01	0.66	0.18	0.87	–0.22	0.81	0.96	0.39
**Non-verbal Intelligence**								
Matrices Task	22.28	1.93	20.69	2.41	17.85	4.16	9.14	4.80 × 10^–4^
Block Task	55.67	9.25	53.48	7.74	40.38	9.00	12.88	3.94 × 10^–5^
Composite	0.50	0.66	0.16	0.56	–0.88	0.94	14.52	1.43 × 10^–5^
**Memory**								
Digit Span Forward	11.28	2.05	11.19	2.01	9.92	1.93	2.02	0.14
Digit Span Backward	9.72	2.44	8.38	2.19	6.54	1.85	7.84	1.2 × 10^–3^
Non-word Repetition Span	27.28	4.62	28.06	2.82	26.62	2.90	0.57	0.57
Counting Span Task	3.56	0.81	3.82	0.82	3.49	0.81	0.68	0.51
Composite	0.16	0.78	0.14	0.70	–0.39	0.57	2.86	0.07
**Attention**								
Auditory Flanker Task	0.01	0.05	0.05	0.06	0.07	0.06	5.51	7.3 × 10^–3^

### Stimuli

Accurate comprehension critically relies on the successful encoding of sentences into vWM as sentences unfold. This information often needs to be retrieved at some later point in time. Later retrieval success indirectly reflects encoding success. Our stimulus set addressed these processes in 128 stimuli. Each stimulus was constituted by an *encoding sentence*, a subsequent *retrieval sentence*, and a follow-up *comprehension question* (Table 2; as described previously by Beese et al., 2017; Vassileiou et al., 2018). In this design, the retrieval of information from the encoding sentence is triggered by the retrieval sentence, while the retrieval success is subsequently assessed by the comprehension question. That is, the comprehension question directly assessed whether participants successfully retrieved the encoded information. In turn, the comprehension accuracy indirectly reflected on encoding success: Correct responses reflected encoding success, whereas incorrect responses reflected encoding failure. This served as the basis to separate the encoding-related oscillatory power into LR and LNR sentences, the comparison of which yields the SME (Paller et al., 1987; Paller and Wagner, 2002; Werkle-Bergner et al., 2006).

**TABLE 2:** Experimental design; examples of encoding sentence, retrieval sentence (requiring retrieval of either subject or object), comprehension question, and feedback [this table is adapted from Beese et al. (2017)].

**Phase**		**Example**	
Encoding (5.0–7.8 s, A)		Der Moderator hat den Schriftsteller und die Sängerin angekündigt und die Moderatorin hat den Schauspieler und die Künstlerin angekündigt. *The presenter (m) had announced the writer (m) and the singer (f) and the presenter (f) had announced the actor (m) and the artist (f).*	

		**Subject**	**Object**

Retrieval (3.5 s, V)		Die sie Ankündigende war nervös. *The one (f) announcing her was nervous.*	Die von ihr Angekündigte war nervös. *The one (f) announced by her was nervous.*

	CORR	War die Moderatorin nervös? *Was the presenter (f) nervous?*	War die Künstlerin nervös? *Was the artist (f) nervous?*
Question (<4 s, V)	GEN-L	War die Sängerin nervös? *Was the singer (f) nervous?*	War die Schauspielerin nervös? *Was the actor (f) nervous?*
	CAT-L	War die Künstlerin nervös? *Was the artist (f) nervous?*	War die Moderatorin nervös? *Was the presenter (f) nervous?*

Feedback (1.0 s, V)		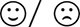	

*f, female; m, male; CORR, correct; GEN-L, gender lure; CAT-L, category lure; A, auditory; V, visual.*

Considering that accurate comprehension relies on successful encoding, it is crucial that the stimulus design discerns memory encoding from language-specific processing efforts. Therefore, in our study, all encoding sentences were of identical syntactic structure while semantic characteristics (e.g., animacy or word frequency) were controlled for. Hence, differences in comprehension accuracy are likely not explained by any language-specific variance, but may instead be related to variance in the encoding success (i.e., LR versus LNR).

The encoding sentences consisted of two conjoined clauses that each contained one subject and two object noun phrases (see Beese et al., 2017; Vassileiou et al., 2018). All nouns were animate, matched for word length (3–5 syllables) and word frequency (frequency class: 9–19; Goldhahn et al., 2012) within and across sentences. To allow for the encoding of an unambiguous cue for later retrieval, both of the two subject noun phrases across the two clauses (e.g., *der Moderator* / *the (male) presenter* and *die Moderatorin/the (female) presenter*) and the object noun phrases within each clause (e.g., first clause: *der Schriftsteller / the (male) writer* and *die Sängerin / the (female) singer*) differed in grammatical gender, which was counterbalanced across stimuli. The specific pairing of two pronouns in the subsequent retrieval sentence unambiguously cued the retrieval of only one specific noun phrase of the encoding sentence. That is, one pronoun referred to only one of the two subjects, while the other pronoun referred to only one of the two objects associated with this subject. For example, in case of an object retrieval, within the retrieval sentence *Die von ihr Angekündigte war nervös* (*The (female) one announced by her was nervous*), *ihr* points to the female subject (i.e., *die Moderatorin / the (female) presenter*) and *die* refers specifically to the female object (i.e., *die Künstlerin / the (female) artist*) associated with this subject. Then, the retrieved noun phrase (i.e., *die Künstlerin / the (female) artist*) can be linked to the adjective (i.e., *nervös/nervous*). Upon the comprehension question, participants needed to match the previously retrieved information with the information presented during the comprehension question. In 50% of all trials, the information presented during the comprehension question (e.g., *War die Künstlerin nervös?* / *Was the (female) artist nervous?*) matched the afore-retrieved correct information (*die Künstlerin / the (female) artist*; CORR). In the other 50%, the information presented during the comprehension question (e.g., *War die Schauspielerin nervös? / Was the actress nervous?*) mismatched the afore-retrieved correct information (*die Künstlerin / the (female) artist*). Thereby, lure questions were introduced to enforce the encoding of category (category lures; CAT-L; 25% of trials) and gender information (gender lures; GEN-L; 25% of trials). Feedback on encoding success was given via a sad or happy emoticon.

To counterbalance the distribution of retrieval cues (i.e., grammatical gender of subject and objects) within and across stimuli, eight variants of each stimulus were constructed. Moreover, counterbalancing gender information as well as retrieval type (i.e., subject or object) yielded four variants of the retrieval sentence. Together, this resulted in 32 combinations of the 128 encoding and retrieval sentences, which were distributed via Latin Square across 32 lists. To match processing demands across participants, retrieval type (i.e., subject or object), answer type (i.e., correct or incorrect), and question type (i.e., correct or lure) were balanced across lists.

To avoid habituation and the development of experimental strategies, each list included an additional 64 filler items (adapted from Meyer et al., 2015). To maximize encoding differences relative to the experimental items, fillers were syntactically more complex (i.e., object relative clauses and topicalization constructions) and cued for biological gender (e.g., *uncle* – *aunt*) instead of grammatical gender. To disguise these differences, syllable count and word frequency were matched to the experimental items. All nouns of experimental and filler items were uniquely used to avoid confounding memory consolidation effects. Stimuli within lists were pseudo-randomized.

### Procedure

Data were collected on 2 days within a single week (mean difference between days = 4.52 days, *SD* = 2.27 days). On the first day, audiometry and neuropsychological testing were carried out always in the same order (i.e., first audiometry, then Vocabulary Task, Similarity Task, Block Task, Matrices Task, Digit Span Forward, Digit Span Backward, Counting Span Task, and Auditory Flankers Task). On the second day, the electroencephalogram (EEG) was acquired, first at rest (Beese et al., 2017) and then during the experimental task. The EEG was recorded in a dimly lit, electrically shielded, soundproof booth. Here we focus on the EEG recording during the experimental task (see also Vassileiou et al., 2018).

Auditory stimuli (i.e., encoding sentences) were presented via headphones (Sennheiser HD202, Sennheiser GmbH & Co., KG, Wedemark, DE). Audio volume was adjusted to 38 dB above the individual hearing threshold (method of limits; Herrmann et al., 2016) to ensure an identical hearing level across participants. Visual stimuli (i.e., retrieval sentence, comprehension question and feedback) were presented in white font (Arial, size = 30 pt) against a gray background on a CRT screen (17^″^, Sony Trinitron Multiscan E220, Sony Corporation, Minato, Japan) using Presentation^®^ (Version 17.0, Neurobehavioral Systems, Inc., Berkeley, CA, United States).

Each trial started with the auditory presentation of the encoding sentence which was followed by the visual presentation of the retrieval sentence, the comprehension question, and the feedback (Table 2). During the encoding sentence, a fixation cross was visually presented and remained on screen until after a jittered time interval of 1.0–1.5 s. Subsequently, the retrieval sentence was visually presented and followed by another jittered pause of 1.5–2.0 s. A comprehension question followed and needed to be answered within 4 s. Participants responded with ‘*yes’* or ‘*no*’ by pressing the button of one of the two single-button response boxes placed individually under their left and right index finger. Button assignment was counterbalanced across participants. After the response, visual feedback was given for 1 s. A jittered inter-trial interval of 1.5–2.0 s completed each trial. After each block of 32 trials, participants were able to take a break (1–4 min).

This procedure of alternating encoding and retrieval phase has increasingly been used in recent years (e.g., Backus et al., 2016; Griffiths et al., 2016), contrary to traditional SME designs that separate encoding and retrieval phase (e.g., Sanquist et al., 1980; Paller et al., 1987; Osipova et al., 2006; Staudigl and Hanslmayr, 2013; Staudigl et al., 2015). The decision to alternate the two phases in our study crucially depended on the high information load of each encoding stimulus. That is, each encoding sentence consisted of 19 words that needed to be encoded as syntactically interrelated semantic information. This degree of information load exceeds that of word pairs (e.g., Sanquist et al., 1980; Haque et al., 2015; Shing et al., 2016) or word-context pairs (e.g., Staudigl and Hanslmayr, 2013; Staudigl et al., 2015; Griffiths et al., 2016). Therefore, the more immediate retrieval of each encoding sentence, in our study, likely resembles SME designs that delay the retrieval phase to after a block of word pairs. This block of word pairs likely constitutes an information load equivalent to that of the encoding sentence in our study.

### Data Acquisition

Electroencephalogram data were acquired from 63 electrodes at a sampling rate of 1,000 Hz within a pass-band from DC to 270 Hz. The setup was referenced against the left mastoid and grounded to the sternum. The vertical and horizontal electrooculogram (EOG) was acquired with bipolar electrodes below and above the right eye as well as at the outer canthi of both eyes, respectively. Scalp electrodes were placed according to the international 10–20 system in an elastic cap (WaveGuard^TM^ original, eemagine GmbH, Berlin, DE) connected to a 72-channel Refa8 amplifier (TMS International B.V., Oldenzaal, Netherlands). Electrode impedances were kept below 5 kΩ.

### Data Analysis

The EEG data were analyzed with MATLAB^®^ (The MathWorks, Inc., Natick, United States), using the FieldTrip Toolbox (Oostenveld et al., 2011). For preprocessing, the data from the encoding sentences were first segmented into epochs ranging from −2 s pre-stimulus onset to 7 s post-stimulus onset. EOG data were discarded. Then, we high-pass filtered the raw data with a zero-phase finite-impulse-response one-pass 1-Hz Kaiser filter (optimal for independent component analysis, ICA; Winkler et al., 2015). The filtered data were re-referenced to the average of all electrodes to remove any noise from the reference electrode. No channels were excluded or interpolated. Afterward, muscle contractions, drifts, and jumps were detected based on a semi-automatic, distribution-based approach. The artifact detection involved temporary filtering (110–140 Hz bandpass, 9th order Butterworth filter) of the data and z-transforming those temporarily filtered data per time point across channels. Any trial with greater *z*-values than 9 at any time point was automatically detected and subsequently visually inspected. All other trials were also visually inspected with respect to their waveform morphology. After this semi-automatic detection, trials that included artifacts were rejected (mean percentage of artifacts rejected = 32.89%, *SD* = 15,63%). For the subsequent ICA, the data were first low-pass filtered to 150 Hz (using a zero-phase finite-impulse-response one-pass Kaiser filter) and down-sampled to 300 Hz. Second, the mean potential at each electrode was subtracted within trials. Third, a principal component analysis was used to reduce the number of dimensions to finally extract 40 independent components (ICs). ICs reflecting vertical and horizontal eye movements as well as heartbeat were detected upon visual inspection of the components’ waveform morphology, power spectrum, and scalp topography (mean number of components rejected = 10.55, *SD* = 1.78 ICs).

While we had high-pass filtered the raw data at 1 Hz for optimal preprocessing (specifically, optimal ICA performance; Winkler et al., 2015), for an optimal data quality in the lower oscillatory frequencies, we chose to use the information that we gained from the preprocessing (i.e., artifact and IC detection) on the raw data filtered at a lower high-pass cutoff value. For this purpose, we high-pass filtered the raw data with a zero-phase finite-impulse-response one-pass 0.1-Hz Kaiser filter and removed artifact trials and ICs from this data set. This 0.1-Hz-filtered data set underwent the same remaining preprocessing steps as mentioned above. Only the 0.1-Hz-filtered data set was used for final data analysis. Afterward, channels A1 and A2 were removed and the data were re-referenced again to the average of all remaining electrodes. Finally, we visually inspected the data one more time for any remaining artifacts, which were removed accordingly; we then subtracted the mean potential at each electrode within trials.

As the task primarily required participants to decode the syntactic information of the noun phrases (NP; i.e., gender and category information thereof), we extracted the six NPs from the overall encoding sentence (each of which expanded on average over 857 ms, *SD* = 144 ms). This resegmentation was performed after the data cleaning of the whole encoding sentence in order to guarantee clean data for all six NPs. The resegmentation was based on the available EEG triggers which were set in close approximation to the NPs. These resulting new segments crucially always included the noun phrase at their core. For instance, the encoding sentence *Der Moderator hat den Schriftsteller und die Sängerin angekündigt und die Moderatorin hat den Schauspieler und die Künstlerin angekündigt (The (male) presenter had announced the (male) writer and the (female) singer, and the (female) presenter had announced the (male) actor and the (female) artist)* was resegmented into “*der Moderator hat/ the (male) presenter had”, “den Schriftsteller und/ the (male) writer and”, “die Sängerin/ the (female) singer”, “die Moderatorin hat/ the (female) presenter had”, “den Schauspieler und/ the (male) actor and” and “die Künstlerin/ the (female) artist”.* This resegmentation created a time series of six NPs per sentence. Crucially, it optimized the statistical analysis as the NPs were now well-aligned across stimuli (for further information on this analysis step, see: Vassileiou et al., 2018).

Using a Hann taper, frequency analysis via a Fast-Fourier Transform was performed upon each NP in steps of 0.5 Hz from 2 to 40 Hz with a spectral smoothing of 1 Hz. As the length of the NPs varied between 492 and 1321 ms (mean = 857 ms; *SD* = 144 ms), equal frequency resolution across all NPs was achieved by zero padding the signal of all NPs to 2 s. After frequency analysis, the signal across all NPs of each sentence position was averaged separately for LN and LNR sentences, yielding six power estimates per condition, per channel–frequency pair and per participant. Subsequently, per participant, these power estimates were baseline corrected relative to a condition-specific pre-stimulus baseline window ranging from −1 to 0 s (corrected signal = (signal – baseline) / baseline). Thereby, the baseline signal was first zero padded to 2 s to match the length of the NPs. Then the baseline signal was averaged across time, per channel-frequency pair, and subtracted from the signal of each NP and then divided by the averaged baseline signal. The baseline signal did not differ between LN and LNR sentences (*z* = 0.31, *p* = 0.76). The choice of baseline correction method is based on our previously published study (Vassileiou et al., 2018), enabling a comparison of results between studies.

### Statistical Analysis

The comprehension accuracy was quantified using d-prime scores (d′) which indirectly indicated encoding success. Compared to traditionally used percentage correct measures, d′-scores have the advantage of controlling for participants’ response bias (i.e., the individual tendency for responding ‘*yes*’ or ‘*no*’). d′ scores were computed by subtracting the z-transformed false alarm rate (FA) from the z-transformed hit rate (H). FA or H of 0 was corrected by 1/N and FA or H of 1 was corrected by (N–1)/N (N = number of trials; Macmillan and Creelman, 2005). In a first step, any participant whose sensitivity to the task was below chance (i.e., *d*′ ≤ 0 and/or accuracy <50%) was excluded from further statistical analyses, as the associated EEG signal cannot be assumed to reflect encoding success. This resulted in the exclusion of 11 from the original 59 participants: 2 younger, 2 middle-aged, and 7 older adults. One additional middle-aged adult was excluded from further statistical analyses as the clean data contained fewer than 10 LNR trials (Scholz et al., 2017).

The comprehension accuracy was used as an index of encoding success. Specifically hits and correction rejections (i.e., correct responses) reflected LR sentences, which indirectly indicated encoding success. In contrast, misses and false alarms (i.e., incorrect responses) reflected LNR sentences, which indirectly indicated encoding failure. This classification deviates from that of previous studies, in which only hits define LR trials and only misses define LNR trials. However, previous designs compared old (i.e., accessible from memory) with new (i.e., inaccessible from memory) items at the retrieval. In contrast, in our design, we contrasted old information that matched the information in the comprehension question with old information that mismatched the information in the comprehension question. Hence, the retrieved information was always accessible in case that it was successfully encoded. Therefore, correct rejections indicate encoding success as much as hits, and false alarms indicate encoding failure as much as misses. On average, participants had 59 LR trials and 26 LNR trials left after artifact rejection, which is comparable to the LR–LNR ratio of previous SME studies (∼70% LR – ∼30% LNR; e.g., Gruber et al., 2004; Hanslmayr et al., 2011; Meeuwissen et al., 2011; Staudigl and Hanslmayr, 2013), guaranteeing above-chance performance as well as roughly comparable numbers of trials per condition. We refrained from bootstrapping LR trials to match the number of LNR trials, as it was previously shown that this would not change the SME (Staudigl and Hanslmayr, 2013).

Conventionally classified frequency bands of theta (4–8 Hz), alpha (8–12 Hz) and beta (15–30 Hz) were adjusted to the individual alpha peak frequency (IAF; for further details, see Beese et al., 2017) because of substantial inter-individual variance of the IAF across age groups (YA mean peak = 10.58 Hz, *SD* = 0.77 Hz; MA mean peak = 10.31 Hz, *SD* = 1.04 Hz; OA mean peak = 9.77 Hz, *SD* = 0.04 Hz; *F*(2,46) = 3.33, *p* = 0.04; Koepruner et al., 1984)—optimizing the interpretation of the frequency bands’ functional significance. The theta band is adjusted as it has been shown to vary as a function of the individual alpha band frequency, dissociating phasic theta synchronization from alpha desynchronization (e.g., Klimesch, 1999), while beta has previously been shown to have a harmonic relationship with alpha (e.g., Carlqvist et al., 2005). We adjusted the theta range from IAF–6 to IAF–2, the alpha range from IAF–2 to IAF+2 and the beta range from IAF+5 to IAF+20 (adapted from Klimesch, 1999). We additionally divided the alpha band into a lower (IAF–2 to IAF) and an upper alpha band (IAF to IAF+2) as Klimesch (1999) has suggested distinct functional relevance of lower and upper alpha. That is, lower alpha (i.e., 8–10 Hz) is associated with attention, while upper alpha (i.e., 10–12 Hz) is related to memory performance.

For sensor-level statistics we averaged the oscillatory power within each frequency band (i.e., for the theta, lower and upper alpha, as well as beta band) across all NPs, within participants. Within each frequency band and electrode, oscillatory power was subjected to a mixed analysis of variance (ANOVA) with the between-subjects factor age group (levels: younger, middle-aged, and old) and the within-subjects factor encoding success (levels: LR and LNR). The variance of oscillatory power at each electrode did not differ between groups (Levene’s test: all 0.009 < *F* > 2.95, all 0.05 < *p* > 0.99). Within frequency bands, across electrodes, *p*-values were FDR-corrected for multiple comparisons. Any interaction effects were dissolved using FDR-corrected simple-effects analyses.

To examine the domain-specificity of the effects *post hoc*, we first correlated the cognitive abilities assessed by the neuropsychological test battery with the sentence comprehension accuracy. To this end, the neuropsychological test scores were first z-transformed and averaged within participants across age groups into composite scores reflecting (see *Standardized neuropsychological measures*): memory (Cronbach’s α = 0.70), non-verbal intelligence (Cronbach’s α = 0.78), verbal intelligence (Cronbach’s α = 0.33) and attention. We then related those cognitive abilities that were associated with sentence comprehension accuracy to age-related SME differences. To this end, we averaged oscillatory power within the lower alpha band across those electrodes for which there were age differences in the SME (see *Results*). Then, separate mixed ANCOVA were computed with the factors age group and encoding success as well as the respective composite score as between-subject covariate. We reasoned that if any of the composite scores relate to the interaction between age group and encoding success, this alpha effect might be additionally explained by other general cognitive functions.

## Results

**FIGURE 1:**
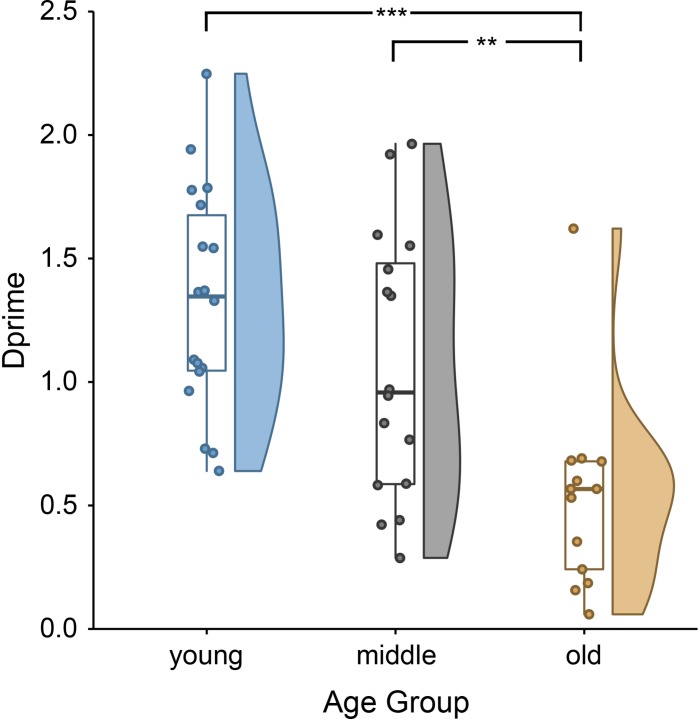
Lower sentence comprehension accuracy, indirectly indicating lower encoding success, for older than younger and middle-aged adults: single subject means (scatter points), group medians (line within boxes), as well as probability density of d-prime values of younger, middle-aged and older adults (for more information on raincloud plots, see Allen et al., 2019). ^∗∗∗^*p* < 0.001 and ^∗∗^*p* < 0.01.

The behavioral data showed clear age differences [*F*(2,46) = 10.88, *p* = 0.0001; Figure 1], with better performance in younger and middle-aged adults compared to older adults. Younger adults remembered on average 73.62% (*SD* = 7.00%) of all sentences correctly (mean d′ = 1.33, *SD* = 0.45). Middle-aged adults remembered 69.03% (*SD* = 8.88%) of all trials correctly (mean d′ = 1.07, *SD* = 0.54). Older adults remembered 60.17% (*SD* = 7.07) of all trials correctly (mean d′ = 0.53, *SD* = 0.39). Group performances exceeded chance level for all age groups (one-sample *t*-test on d′ within age group; all *t* > 4.88, all *p* < 0.001).

**FIGURE 2:**
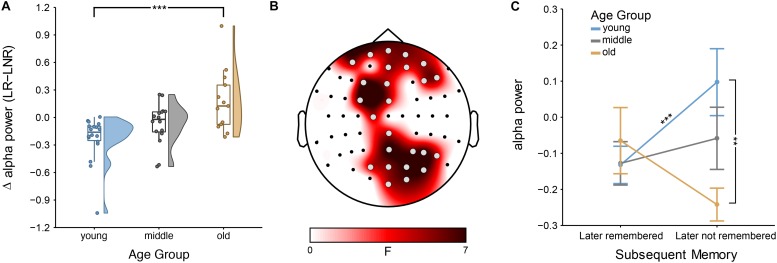
Subsequent memory effect (SME) across age groups: **(A)** Encoding-related power decreases in younger adults [i.e., lower power for later-remembered (LR) than for later-not-remembered (LNR) sentences], averaged across all significant electrodes (shown in **B**) within the lower alpha band (i.e., individual alpha peak frequency (IAF) – 2 Hz to IAF) are attenuated in middle-aged adults and turn into a power increases in older adults; error bars reflect one standard error; ^∗∗∗^*p* < 0.001 **(B)** Scalp topography shows that the age difference in the SME within the lower alpha band is distributed across bilateral frontal and right-hemispheric parietal sites (gray electrodes: *p* < 0.05, FDR-corrected), **(C)** Interaction effect between age groups and encoding success: lower alpha power differs between younger and older adults for LNR but not LR sentences; ^∗∗∗^*p* < 0.001 and ^∗∗^*p* < 0.05.

The mixed ANOVAs at each electrode showed an age dependence of the SME for the lower alpha band—but not for the theta, upper alpha or beta band (see Supplementary Figures S1, S2 for further details on theta and beta, respectively). Specifically, the interaction effect between age group and encoding success was significant at bilateral frontal and right-hemispheric parietal sites (all 4.30 < *F*(2,44) < 7.44; all 2.13 × 10^–2^ < *p* < 4.79 × 10^–2^, FDR-corrected; all 0.03 < η^2^ < 0.08; Figure 2). There was no main effect of age [all 0.06 < *F*(2,44) < 3.51; all 7.36 × 10^–1^ < *p* < 9.38 × 10^–1^, FDR-corrected] or of encoding success [all 0.01 × 10^–5^ < *F*(1,44) < 8.80; all 2.95 × 10^–1^ < *p* < 9.21 × 10^–1^, FDR-corrected].

**FIGURE 3:**
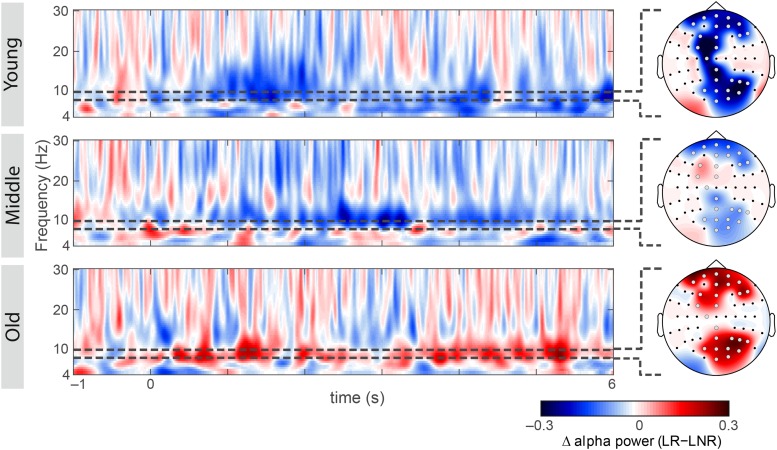
Subsequent memory effect within each age group at electrodes of group-level significance (as shown in Figure 2B): oscillatory power differences between later-remembered (LR) than for later-not-remembered (LNR) sentences across the theta, alpha and beta frequency range (4–30 Hz) across the minimum sentence length, including the baseline window (–1 to 6 s) for younger, middle-aged and older adults; scalp topographies showing encoding-related lower alpha power differences between LR and LNR (gray electrodes indicate group-level significance). All spectra and topographies were adjusted for the individual alpha peak.

Simple-effects analyses revealed consistent age differences between younger and older adults in the overall SME (i.e., LR–LNR; all 3.55 × 10^–4^ < *p* < 1.51 × 10^–2^, FDR-corrected; Figure 2A) at all electrodes for which the interaction effect between age group and encoding success was significant (Figure 2B). At a closer look, age-related SME differences between younger and older adults were actually only related to age differences in the alpha power underlying LNR but not LR sentences (at F3, FC3, P6, PO4, PO6, PO8 and O2; all 3.85 × 10^–3^ < *p* < 1.01 × 10^–2^, FDR-corrected; Figure 2C). Within age groups, only younger adults showed a significant difference in encoding-related oscillatory power (i.e., LR–LNR; all 4.87 × 10^–5^ < *p* < 3.13 × 10^–2^, FDR-corrected; at FP2, AF8, Fz, F3, FC3, FCz, C1, CPz, Pz, P4, P6, P8, POz, PO4, PO8 and O2; Figure 3). Specifically, younger adults showed a negative SME (i.e., LR < LNR). This effect was numerically, though not significantly, attenuated in the middle-aged adults. This turned into a numerical, though not significant, positive SME in the older adults (i.e., LR > LNR).

*Post hoc*, we found that sentence comprehension was associated both with working memory (*r* = 0.52, *p* = 3.75 × 10^–4^, FDR-corrected) and non-verbal intelligence (*r* = 0.52, *p* = 3.75 × 10^–4^, FDR-corrected) across age groups. Both, working memory and non-verbal intelligence differed across age groups (see Table 1). Therefore, further *post hoc* analyses were conducted to test the domain-specificity of age-related SME differences. The results revealed that while both working memory and non-verbal intelligence associated with sentence comprehension, age differences therein did not associate with age-related SME differences [all 0.04 < *F*(2,41) < 5.08; all 0.27 < *p* < 0.96; FDR-corrected].

## Discussion

This study gives first indications that older adults’ sentence comprehension difficulties may be related to age differences in oscillatory patterns underlying sentence encoding. We found encoding-related age differences between younger, middle-aged, and older adults in lower alpha-band power. Specifically, comparing encoding-related alpha power between LR and LNR sentences, younger adults displayed a negative SME (i.e., LR < LNR), which was attenuated in the middle-aged adults and shifted toward a positive SME in older adults (i.e., LR > LNR). Age differences in the SME were dominated by differences between younger and older adults in the power underlying LNR, but not LR sentences. That is, when older adults failed to encode sentences, the oscillatory pattern was reversed; but when older adults successfully encoded sentences, the oscillatory pattern matched that of younger adults. We tentatively suggest that age-related sentence comprehension difficulties are associated with age differences in sentence encoding.

We here found sentence comprehension difficulties to be related to age differences in the alpha band, but not the theta and beta bands. In younger adults alpha band power has previously been shown to decrease as syntactic information is encoded into vWM (Bastiaansen et al., 2009; Bonhage et al., 2017; Vassileiou et al., 2018). Such alpha power decreases have specifically been related to the success of encoding in younger adults (e.g., Otten et al., 2001; Schott et al., 2002; Hanslmayr and Staudigl, 2014). Here, we also found an alpha power decrease when younger adults successfully encoded sentences into vWM, which likely enabled them to accurately comprehend sentences. Difficulties in sentence comprehension emerged as the encoding success became gradually linked to alpha power increases in old age. In contrast to modulations of alpha-band power by variations in memory encoding demands, increased theta power during language comprehension has been linked to retrieval operations both on working memory and long-term memory (e.g., Bastiaansen et al., 2002b, 2009; Haarmann and Cameron, 2005; Meyer et al., 2015), while decreased beta power was associated with semantic and potentially predictive processing (e.g., Weiss and Rappelsberger, 1996; Wang et al., 2012; Lewis and Bastiaansen, 2015). However, in this study, sentence comprehension difficulties in older adults did not relate to age differences in either theta or beta and may hence not relate to any semantic processes. Instead, in our study, sentence comprehension difficulties related to age differences the alpha band and may hence be associated with age differences in the encoding of syntactic information. This finding is in line with previous studies showing that specifically syntactic but not semantic processing is compromised at old age (e.g., Stine, 1990; Beese et al., 2019; Poulisse et al., 2019).

With alpha band power having been previously related to inhibitory processes, the lifespan shift from an alpha-band power decrease to an alpha-band power increase may reflect a functional shift from cortical disinhibition to inhibition (e.g., Jensen and Mazaheri, 2010). While increased alpha-band power has been proposed to inhibit task-irrelevant regions, decreased alpha-band power is supposed to gate the information flow toward task-relevant regions (Klimesch et al., 2007; Haegens et al., 2010; Jensen and Mazaheri, 2010). Following this train of thought, alpha-band oscillations are often thought to regulate the information flow through the cortex. Specifically, task-irrelevant information is suppressed when alpha power increases (Palva et al., 2005; Sauseng et al., 2009; Zanto and Gazzaley, 2009; Jensen and Mazaheri, 2010; Hanslmayr et al., 2011), while task-relevant information is enhanced when alpha power decreases (Babiloni et al., 2004; Hartmann et al., 2012; Hauck et al., 2015). Therefore, successful encoding in younger adults may be supported by the enhancement of task-relevant information through cortical disinhibition, as reflected by decreased alpha power. Older adults’ difficulties in the processing of vMW-taxing sentences may be associated with an age-related inefficiency in cortical disinhibition.

As sentences unfold, upcoming information interferes with already encoded information (Lewis et al., 2006). The ability to inhibit interfering information is thus a critical determinant of successful sentence comprehension (e.g., Lewis et al., 2006; Van Dyke, 2007; Glaser et al., 2013; Santi et al., 2015). Age deficits in this ability to inhibit interfering information may compromise sentence processing (Hasher and Zacks, 1988). In support, in our study, when sentences were later-not-remembered (i.e., LNR sentences), alpha power was found lower in older than younger adults. Linking alpha power to inhibition, this may reflect inhibitory deficits in older adults. In contrast, when sentences were later remembered (i.e., LR sentences), alpha power did not differ between age groups. Hence, age-related SME differences may be predominantly driven by encoding failure (i.e., LNR sentences) rather than encoding success (i.e., LR sentences). However, when comparing encoding success to encoding failure, it appears that younger and older adults rely on opposing processes (i.e., negative versus positive SME). That is, in comparison to encoding failure, younger adults may achieve encoding success through cortical disinhibition (i.e., alpha power LR < LNR). In contrast, older adults may achieve encoding success through cortical inhibition (i.e., alpha power LR > LNR). Hence, successful encoding may rely on a disinhibition-to-inhibition shift across the lifespan. In this vein, previous studies showed that older adults remain able to enhance task-relevant information (Gazzaley et al., 2005; Geerligs et al., 2014), but fail to inhibit interfering information (Radvansky et al., 2001; Gazzaley et al., 2008). Such a decrease in inhibition allows for increased distractibility (Lavie et al., 2004) which overloads older adults’ limited vWM capacity storage (Vogel et al., 2005). This in turn affects their memory performance (Eriksen and Eriksen, 1974; Gazzaley et al., 2005; Zanto and Gazzaley, 2009). Therefore, a lifespan shift from disinhibition-to-inhibition may be a mechanistic substrate of age differences in sentence encoding underlying age-related sentence comprehension difficulties.

This lifespan shift from disinhibition to inhibition may also indicate an age-related shift from bottom-up to top-down processing. That is, disinhibiting detailed sentence information may imply a bottom-up extraction of such information. In contrast, inhibiting the information may imply a top-down extraction of fewer details, focusing on gist information. While our results suggest younger adults’ encoding success to be related to the disinhibition of information, older adults’ encoding success may be linked to the inhibition of information. Accordingly, lower alpha power underlying encoding success has been associated with bottom-up encoding in younger adults (Hanslmayr et al., 2009). In contrast, older adults have been shown deficient in bottom-up processing (Wingfield et al., 1991; Madden et al., 2005; Madden, 2007). In line with this, older adults also show greater alpha power related to encoding success than younger adults (Karrasch et al., 2004). In addition, it has been noted that older adults do not extract rich and detailed information, but rather the overall gist of sentences when sentences were accurately comprehended (Tun et al., 1998; Christianson et al., 2006). Together with our results this may suggest that older adults can successfully encode sentences through top-down processing (Wingfield et al., 1991; Whiting et al., 2007, 2014). However, in our study, the task required a bottom-up, word-by-word encoding of detailed syntactic information; top-down encoding would have been insufficient to successfully solve the task at hand. This may explain our observation that older adults showed lower performance accuracy.

One might expect that age differences in sentence encoding may be associated with age differences in domain-general cognitive abilities that are known to support sentence comprehension (Fedorenko, 2014; Beese et al., 2017; Hoffman and Morcom, 2018). That is, an age-related decrease in vWM capacity (e.g., Bopp and Verhaeghen, 2005) or attention (e.g., Madden et al., 2005; Madden, 2007) may link to the employment of gist extraction in older adults (Tun et al., 1998; Christianson et al., 2006; Ferreira and Patson, 2007). While our *post hoc* analyses showed that sentence comprehension is generally supported by domain-general cognitive abilities, we did not observe any relation between those domain-general cognitive abilities and the encoding-related oscillatory differences between age groups.

## Limitations

One obvious limitation of this study is the small sample size of the individual age groups. However, our previously published study (Vassileiou et al., 2018) showed that the SME effect for this design is already reliable at a small sample size, that is, 22 participants [effect size (*dz*) = −0.81], as estimated by a power analysis (Faul et al., 2009). However, after data cleaning the current data set includes only 18 younger, 16 middle-aged and 13 older adults. Even though the effects of this study are likely underpowered, they contribute to the field by providing a tentative link between age-related sentence comprehension difficulties and encoding-related electrophysiological dynamics. Future studies should include a sufficient number of participants in each group, focusing on younger and older adults only, as the current effect was linear across the lifespan.

Moreover, another concern regards the signal-to-noise ratio (SNR). The SNR of encoding-related activity decreases across the lifespan with diminishing retrieval confidence (e.g., Friedman and Trott, 2000; Gutchess et al., 2007; Cansino et al., 2010; Kamp and Zimmer, 2015). High retrieval confidence is associated with recollection-based memory (i.e., “remember” responses; Tulving, 1985) while lower retrieval confidence is associated with familiarity-based memory (i.e., “know” responses; Brewer et al., 1998; Friedman and Trott, 2000). Older adults produce fewer recollection-based and equal or more familiarity-based responses compared to young adults (e.g., Mark and Rugg, 1998; Friedman and Trott, 2000; Friedman et al., 2007). Previous studies found age differences in the encoding-related signal primarily when the information was retrieved with high confidence. That is, at high confidence, the magnitude of the SME is reduced in older compared to younger adults (Friedman and Trott, 2000; Gutchess et al., 2007; Kamp and Zimmer, 2015) while also onset and topography differs across age groups (Cansino et al., 2010). Such age-related SME differences may disappear when high and low retrieval confidence is not discerned (Gutchess et al., 2007). As we did not assess retrieval confidence, the SNR in our data may be reduced in older compared to younger adults. This may be the reason why encoding-related differences between LR and LNR were just numerical and not significant in older adults while the SME was clearly significant in younger adults.

## Conclusion

Our study contributes to the understanding of neurocognitive aging, especially in the field of sentence processing. We here addressed the question whether age-related sentence comprehension difficulties associate with age differences in the neural correlates underlying sentence encoding. The results provide initial evidence for a lifespan shift from decreased to increased encoding-related alpha power, which likely reflects a shift from cortical disinhibition to inhibition. That is, in comparison to encoding failure, encoding success is achieved through decreased alpha power in younger adults, reflecting cortical disinhibition, and increased alpha power in older adults, reflecting cortical inhibition. Disinhibition may entail bottom-up information processing while inhibition may entail top-down information processing. Overall, our results suggest that age-related sentence comprehension difficulties are not only language-specific but may also associate with memory encoding-related electrophysiological alternations across the lifespan.

## Data Availability

The datasets generated for this study are available on request to the corresponding author.

## Ethics Statement

The protocol of this study was approved by the ethics committee of the University of Leipzig and was conducted in accordance with the Declaration of Helsinki with written informed consent from all subjects.

## Author Contributions

All authors have planned and designed this study. CB and BV collected the data. CB analyzed the data. CB, LM, and AF wrote the manuscript.

## Conflict of Interest Statement

The authors declare that the research was conducted in the absence of any commercial or financial relationships that could be construed as a potential conflict of interest.
