# Supplementary material for: Age Differences in Encoding-Related Alpha Power Reflect Sentence Comprehension Difficulties
Source: Front Aging Neurosci. 2019 Jul 17;11:183. doi: 10.3389/fnagi.2019.00183 (PMC6654000; doi:10.3389/fnagi.2019.00183)

Supplementary Material

1 Supplementary Figures

Figure S1 A subsequent memory effect (SME) was not present within the theta band (i.e., individual alpha peak frequency (IAF) – 6 Hz to IAF – 2 Hz) for any age group: (A) Encoding-related power did not differ between later-remembered (LR) and later-not-remembered (LNR) sentences for younger, middle-aged or older adults (here power was averaged across all electrodes; error bars reflect one standard error), (B) Scalp topography also shows that there are no age differences in the SME, (C) There were no age differences in either LR or LNR.

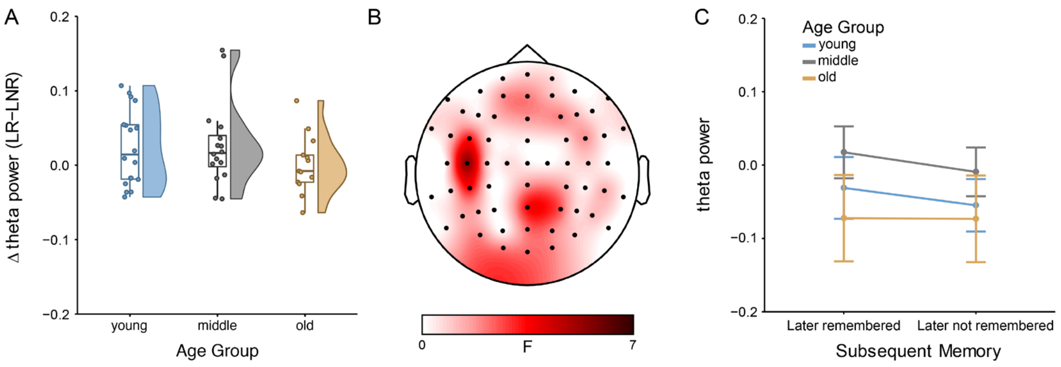

Figure S2 A subsequent memory effect (SME) was not present within the beta band (i.e., individual alpha peak frequency (IAF) + 5 Hz to IAF + 20 Hz) for any age group: (A) Encoding-related power did not differ between later-remembered (LR) and later-not-remembered (LNR) sentences for younger, middle-aged or older adults (here power was averaged across all electrodes; error bars reflect one standard error), (B) Scalp topography also shows that there are no age differences in the SME, (C) There were no age differences in either LR or LNR.

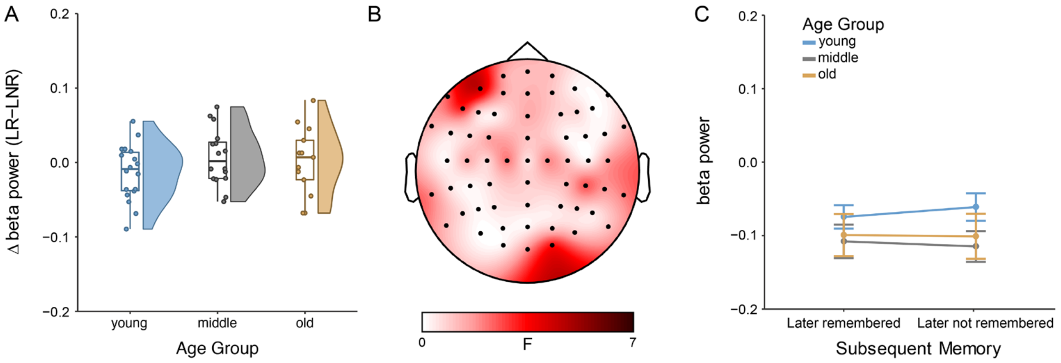

Supplement: Supplementary file 1 [file Data_Sheet_1.pdf]
